# Arginase-1 targeting peptide vaccine in patients with metastatic solid tumors – A phase I trial

**DOI:** 10.3389/fimmu.2022.1023023

**Published:** 2022-10-17

**Authors:** Cathrine Lund Lorentzen, Evelina Martinenaite, Julie Westerlin Kjeldsen, Rikke Boedker Holmstroem, Sofie Kirial Mørk, Ayako Wakatsuki Pedersen, Eva Ehrnrooth, Mads Hald Andersen, Inge Marie Svane

**Affiliations:** ^1^ National Center for Cancer Immune Therapy (CCIT-DK), Department of Oncology, Copenhagen University Hospital, Herlev, Denmark; ^2^ IO Biotech ApS, Copenhagen, Denmark

**Keywords:** arginase-1, peptide, vaccination, solid tumors, first-in-human

## Abstract

**Background:**

Arginase-1-producing cells inhibit T cell-mediated anti-tumor responses by reducing L-arginine levels in the tumor microenvironment. T cell-facilitated elimination of arginase-1-expressing cells could potentially restore L-arginine levels and improve anti-tumor responses. The activation of arginase-1-specific T cells may convert the immunosuppressive tumor microenvironment and induce or strengthen local Th1 inflammation. In the current clinical study, we examined the safety and immunogenicity of arginase-1-based peptide vaccination.

**Methods:**

In this clinical phase I trial, ten patients with treatment-refractory progressive solid tumors were treated. The patients received an arginase-1 peptide vaccine comprising three 20-mer peptides from the ARG1 immunological “hot spot” region in combination with the adjuvant Montanide ISA-51. The vaccines were administered subcutaneously every third week (maximum 16 vaccines). The primary endpoint was to evaluate safety assessed by Common Terminology Criteria for Adverse Events 4.0 and laboratory monitoring. Vaccine-specific immune responses were evaluated using enzyme-linked immune absorbent spot assays and intracellular cytokine staining on peripheral blood mononuclear cells. Clinical responses were evaluated using Response Evaluation Criteria in Solid Tumors 1.1.

**Results:**

The vaccination was feasible, and no vaccine-related grade 3–4 adverse events were registered. Nine (90%) of ten patients exhibited peptide-specific immune responses in peripheral blood mononuclear cells. Six (86%) of the seven evaluable patients developed a reactive T cell response against at least one of the ARG1 peptides during treatment. A phenotypic classification revealed that arginase-1 vaccine-specific T cells were both CD4+ T cells and CD8+ T cells. Two (20%) of ten patients obtained stable disease for respectively four- and seven months on vaccination treatment.

**Conclusion:**

The peptide vaccine against arginase-1 was safe. Nine (90%) of ten patients had measurable peptide-specific responses in the periphery blood, and two (20%) of ten patients attained stable disease on protocol treatment.

**Clinical trial registration:**

https://clinicaltrials.gov/ct2/show/NCT03689192, identifier NCT03689192.

## Introduction

The search for new cancer therapies has led to major breakthroughs within the past decade. However, more treatment options for patients with metastatic solid tumors are needed, and the current standard therapies often induce substantial side effects (1). Although immunotherapeutic agents such as checkpoint inhibitors (CPIs) have revolutionized the treatment of some cancer types, there is still an unmet need for tolerable and effective treatments for many patients with metastatic solid tumors, especially patients with non-inflamed tumors (2, 3).

Cancer immunotherapy is based on principles of immune surveillance. Tumor cells often escape immune recognition, and the enzyme arginase-1 (ARG1) plays an important role in tumor-mediated immune suppression (4). ARG1 hydrolyses the amino acid L-arginine to ornithine and urea. In the liver, this process is essential to ammonia detoxification (5). In a tumor setting, an ARG1-induced depletion of L-arginine impairs T cell receptor (TCR) signaling through the downregulation of TCR ζ chain expression, induces T cell cycle arrest, limits T cell differentiation, and reduces cytokine production (6). As a result, increased ARG1 expression dampens T cell-mediated anti-tumor responses in the tumor microenvironment. Tumor cells can promote the differentiation of ARG1-expressing cells such as tumor-associated macrophages (TAMs) and myeloid-derived suppressor cells (MDSCs) to evade immune surveillance (7).

ARG1 is primarily a liver-specific enzyme, but the protein is also highly expressed in several human cancers, including lung, ovarian, renal, breast, and head and neck (2, 8–11). ARG1 expressing TAMs and MDSCs are major players in the induction of immunosuppressive microenvironments associated with many of these tumors. These lesions were recently defined as tumors with an “excluded” phenotype because they excluded CD8+ T cells from the tumor parenchyma (12). We have recently found spontaneous effector T cell reactivity against ARG1 peptides in peripheral blood mononuclear cells (PBMCs) of both cancer patients and healthy donors at the National Center for Cancer Immune Therapy (CCIT-DK). We further demonstrated that these T cells recognize and react against DCs in addition to B cells expressing ARG1 (13).

Additionally, we have shown that these pre-existing T cells responses against ARG1 are part of the T-cell memory repertoire (14). Especially one “hot spot” region within the ARG1 protein consists of frequently recognized T cell-stimulating epitopes. Both CD4+ and CD8+ ARG1 specific T cells were observed, indicating the existence of both HLA class I and II epitopes in the ARG1 peptide “hot spot” region (13).

Immune modulatory vaccination is a novel unconventional way to target immune suppressive cell populations in the tumor microenvironment (15). The role of ARG1 in tumor-mediated immune suppression makes it a promising therapeutic target for immune-modulatory vaccines. In contrast to the other clinical strategies that target TAMs or MDSCs, this combines TAM depletion through direct killing by cytotoxic T cells and TAM reprogramming by introducing pro-inflammatory cytokines into the immunosuppressive microenvironment (16). Based on the previous pre-clinical and clinical vaccination trials targeting proteins involved in immune regulation from CCIT-DK (13, 14, 16–19), we planned a trial with a therapeutic ARG1 peptide vaccine for patients with high MDSC-expressing cancers treated at the Oncology Department at Herlev Hospital. The primary endpoint was to examine the safety and feasibility of the vaccination treatment (20–22). The overall purpose was to activate ARG1-specific T cells to target the tumor microenvironment, strengthen or induce local Th1 inflammation, and potentially eliminate or reprogram ARG1-expressing immune suppressive cells.

In this phase I study, we vaccinated ten patients with three 20-mer peptides from the ARG1 “hot spot” region and the adjuvant Montanide ISA-51.

## Materials and methods

### Trial design

The trial was a phase I, investigator-initiated, single-armed, and open-label study. Patients were evaluated and treated at the Department of Oncology, Copenhagen University Hospital, Herlev, Denmark, between January 2019 and December 2021. The ARG1 vaccine comprising ARG1 peptide in Montanide ISA-51, and was administered subcutaneously every third week for up to 48 weeks (16 vaccines in total). Patients who were not excluded from the trial due to progression were scheduled for three and six months follow-up evaluations after the last vaccine. The trial was closed on January 19^th^, 2022, three weeks after the last patient was excluded. The Data cut-off was April 1^st^, 2022.

The primary objective was to evaluate the vaccination feasibility and safety according to Common Terminology Criteria for Adverse Events (CTCAE) 4.0. The secondary objectives were to evaluate immunomodulatory characteristics and clinical efficacy consistent with Response Evaluation Criteria in Solid Tumors (RECIST) 1.1.

The study was conducted as stated in the Declaration of Helsinki, following Good Clinical Practice (GCP) recommendations, and monitored by the GCP unit in Copenhagen, Denmark. The phase I trial was approved by the Ethics Committee for the Capital Region, Denmark, and The Danish Medicines Agency; EudraCT no: 2018-000719-26. Clinical trial registration at www.clinicaltrials.gov with identification NCT03689192.

### Vaccine

Each vaccine consisted of 300 μg ARG1: a combination of three 20-amino acid peptides with the sequences: AKDIVYIGLRDVDPGEHYIL (ARG1-18), DVDPGEHYILKTLGIKYFSM (ARG1-19), and KTLGIKYFSMTEVDRLGIGK (ARG1-20) (PolyPeptide, France). 100 μg of each ARG1 peptide was dissolved in sterile water, filtered, and frozen at −20°C in NUNC™ CyroTubes™ CryoLine System Internal Thread, Sigma-Aldrich. A maximum of two hours before administration, peptides were thawed for injection. Shortly before injection, the dissolved peptides were emulsified 1:1 with the adjuvant Montanide ISA-51 (SEPPIC) to a volume of 1 ml. The vaccines were administered subcutaneously every three weeks, repeatedly until reaching a total of 16 vaccines.

### Patients

Patients above 18 years of age with advanced solid tumors, including non-small cell lung cancer (NSCLC), colorectal cancer, urothelial cancer, breast cancer, ovarian cancer, malignant melanoma, and squamous cell carcinoma of the head and neck (HNSCC) were included regardless of prior oncological treatment. The patients were included at the Department of Oncology, Copenhagen University Hospital, Herlev, Denmark. The main inclusion criteria were: progressive or recurrent disease on or following treatment with standard of care agents including chemotherapy and/or checkpoint inhibitors, Eastern Cooperative Oncology Group (ECOG) Performance Status (PS) of 0–1, a life expectancy of at least three months, mandatory provision of archival blood for biomarker testing at baseline, at least one measurable target lesion consistent with RECIST 1.1. The main exclusion criteria were severe comorbidities, treatment with systemic corticosteroids >10 mg/day prednisone or equivalent within three weeks prior to randomization, active autoimmune disease, and concurrent treatment with agents that interfere with the urea cycle (Valproate or Xanthine Oxidase inhibitors). All Patients were enrolled following oral and written informed consent.

### Clinical evaluation

Adverse events were assessed by (CTCAE) 4.0 and laboratory monitoring. Biomedical markers were evaluated prior to inclusion and before every vaccine. Clinical responses were assessed every three months until progression by standard radiologic imaging with CT, PET-CT, or MR scan, depending on the individual cancer diagnosis and prior imaging evaluation methods. Treatment responses were evaluated according to RECIST 1.1, and objective responses were categorized as complete response (CR), partial response (PR), stable disease (SD), or progressive disease (PD). Patient data were registered in the eCRF program REDCap.

### Processing project blood samples

Project blood samples were obtained from all patients at baseline and following every evaluation scan. Blood samples were handled within five hours after collection. Peripheral blood mononuclear cells (PBMCs) were isolated with Lymphoprep (Medinor) separation. Isolated PBMCs were counted on the analyzer (Sysmex XP-300) and frozen in 90% Human Serum with 10% DMSO (Sigma Aldrich) using controlled-rate freezing (Cool-Cell, BioCision) in a −80°C freezer. The samples were transferred to - 140°C the following day.

### ELISPOT assay

ARG1-specific T cell responses were assessed using indirect interferon (IFN)-γ enzyme-linked immunospot (ELISpot) assay. PBMCs from the treated patients were stimulated with ARG1 20-mer peptides and low-dose IL-2 (120 U/ml) *in vitro*. The cells were stimulated for 14 days before IFN-γ ELISpot assays with 2.8-3 x 10^5^ cells per well and transferred to a 96-well, PVDF ELISpot plate (membrane-bottomed), (MultiScreen MSIPN4W50, Millipore) with a precoating of the antibody IFN-γ-capture (1-D1K clone, Mabtech). Five 5 μM of diluted ARG1 peptide and DMSO stocks were added, and the corresponding DMSO dose was added to the control wells. The majority of the samples were set up in triplicates for peptide and control stimulations using PBMCs from the individual patients. Duplicates or singlets were set up for PBMC samples from patient AA1809.09 due to poor cell recovery.

The cells were incubated with the peptides in ELISpot plates for 16–18 hours. The plates were then washed, and the biotinylated secondary antibody anti-IFN-γ mAb (7-B6-1, Mabtech) was included. The unbound anti-IFN-γ mAb was washed off after a two-hour incubation time. Streptavidin-conjugated alkaline phosphatase (Mabtech) was then added for one hour, and the unbound was washed off. Lastly, the BCIP/NBT substrate (Mabtech) was then added.

Spots were counted using the ImmunoSpot S6 Ultimate V analyzer (CTL Analyser). Responses were found by calculating the variance between the average spots-numbers in the wells stimulated with ARG1 peptide and the control wells. Vaccine-specific responses were defined as accurate if there was a statistically significant variance between the spot count in the peptide-stimulated wells and the control wells according to distribution-free resampling (DFR) statistical analysis as stated by Moodie et al. (23). The spot count in the wells with peptide stimulation had to be twice the spot count in the control wells for both duplicates and singlets.

### Intracellular cytokine staining of PBMCs

ARG1 peptide-specific T cell response phenotype was tested by intracellular cytokine staining (ICS) of PBMCs stimulated with the individual 20-mer peptides. Similar to ELISPOT assay, prior to being used in the ICS assay, PBMCs were stimulated *in vitro* with the individual 20-mer peptides and low dose IL-2 (120U/ml) for 14 days. After 14-day culture, PBMCs were stimulated with 5 μM of the appropriate ARG1 peptide or for five hours in a 96-well plate. One hour after adding the peptide, the protein transport inhibitor BD GolgiPlug™ (BD Biosciences) was added. Non-stimulated PBMCs were used as a control to determine the background cytokine levels. Following the 5-hour incubation, the PBMCs were stained using the following antibodies: CD3-APC-H7 (BD Biosciences), CD4-PerCP (BD Biosciences), CD8- FITC (BD Biosciences). The dead cells were stained with FVS510 (BD Biosciences). Stained samples were then fixed and permeabilized overnight using eBioscience™ Fixation/Permeabilization buffers (eBioscience) as stated by the instructions of the manufacture. The following day cells were stained intracellularly using the eBioscience permeabilization buffer (eBioscience) with TNFα-BV421 (BD Biosciences) and IFNγ-APC (BD Biosciences). The samples were analyzed using the FACSCanto™ II (BD Biosciences) with BD FACSDiva software (v. 8.0.2).

### Statistical analysis

ELISPOT assay responses were determined using the DFR method (23). Survival curves were calculated in GraphPad Prism version 9.0.0 using the Kaplan–Meier method. To compare responses to the ARG1 peptides, the Wilcoxon matched-pairs signed-rank test was used. P values ≤ 0.05 were definite as significant. Safety was evaluated according to CTCAE 4.0, and the adverse events are listed in **Table 2**. No statistical analyses were applied.

## Results

### Patient baseline characteristics and treatment

**Figure 1:**
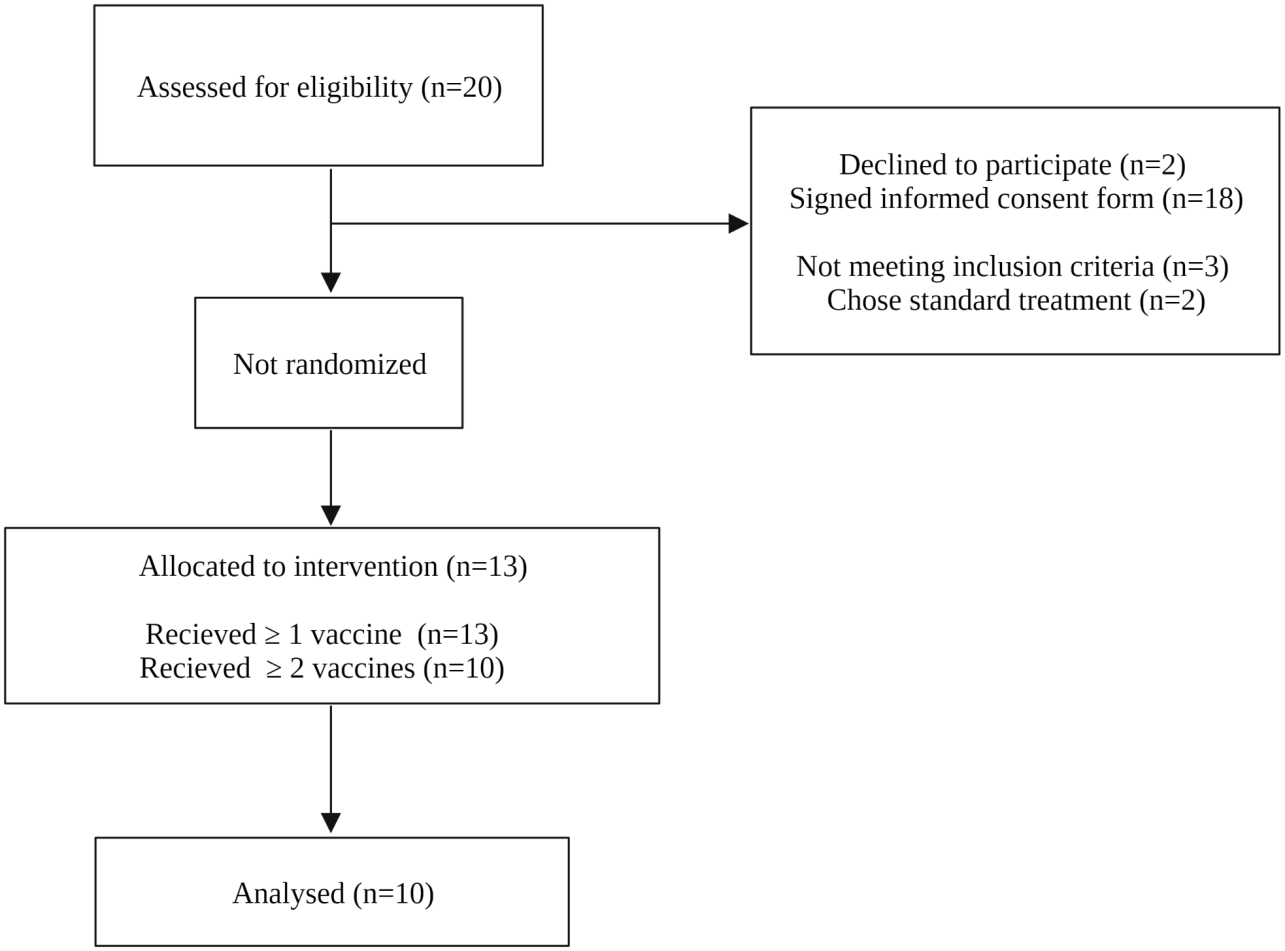
Consort diagram. Twenty patients with metastatic solid tumors were assessed for eligibility. Thirteen patients were enrolled and received the study treatment. Three patients received < 2 vaccines and were replaced with new participants. Ten patients received ≥ 2 vaccines and were evaluated.

Thirteen patients with progressive metastatic colorectal cancer, NSCLC, urothelial cancer, breast cancer, ovarian cancer, malignant melanoma, or HNSCC on- or following treatment with standard of care agents were enrolled and treated with the study therapy. The selected diagnoses were based on cancers associated with increased circulating- or tumor infiltrating MDSCs (20–22). The included patient group was further restricted to the cancers treated at the Oncology Department at Herlev Hospital. The median age of the clinical study participant was 67 years. Ovarian cancer was the most frequent diagnosis among the evaluable patients (n=3). All patients had advanced disease and progressed on several treatment lines before enrolment. Baseline characteristics are listed in **Table 1**. Three patients received < 2 vaccines before exclusion due to rapid disease progression. The three patients were replaced with new participants as specified per protocol. Ten patients received ≥ 2 vaccines and were considered evaluable (**Figure 1**). At the end of trial in January 2022, all ten patients were excluded due to either clinical cancer progression or cancer progression according to RECIST 1.1. The median number of vaccinations for the ten evaluable patients was 4.75 (ranging from 2─12 vaccines). Four patients received subsequent therapy following progression on study treatment. Vaccine production and administration were evaluated as feasible procedures.

**Table 1:** A list of patients treated.

Baseline characteristics, N=10									
**Patient ID**	**Cancer**	**Age**	**Sex**	**Baseline LDH**	**Prior therapy lines**	**BOR to most recent prior treatment**	**ARG1 vaccines**	**Post ARG1 therapy lines**	**Metastatic sites**
7	Breast cancer	67	F	564↑	5	PD	4	0	Lymph nodes, liver, adrenal gland, and lungs
15	Breast cancer	58	F	256↑	11	PD	3	0	Lymph nodes, liver, lungs, breast, bones, skin, and kidney
1	Colon cancer	75	F	1020↑	3	PD	2	0	Lung and liver
2*	Colon cancer	66	F	521↑	3	PD	1	0	Lungs, liver, and peritoneal carcinomatosis
8*	Colon cancer	53	M	314↑	4	SD	1	1	Lungs, liver, and bones
10	Malignant melanoma	81	M	206	4	SD	12	0	Lymph nodes, thorax wall, and adrenal gland
12	Malignant melanoma	56	F	174	3	SD	3	0	Lymph nodes, lung, and bones
3*	Malignant melanoma	58	M	523↑	8	PD	1	0	Lymph nodes, subcutis, muscles, breasts, adrenal gland, bones, and peritoneal carcinomatosis
9	Ocular melanoma	73	F	202	2	SD	6	1	Liver
4	Ovarian cancer	66	F	241↑	7	PD	4	2	Lymph nodes and peritoneal carcinomatosis
13	Ovarian cancer	77	F	465↑	4	SD	4	2	Liver
14	Ovarian cancer	66	F	209↑	2	PD	3	0	Lymph nodes and peritoneal carcinomatosis
16	Rectal cancer	67	F	229↑	2	SD	3	1	Lungs and liver

*Patients receiving less than two vaccines were excluded and replaced with new participants. ↑ indicates elevated LDH levels. The definition of elevated LDH is age depended: 18-69 years: >205 U/L; 70-125 years: >255U/L. ARG1, arginase 1; BOR, best overall response; LDH, lactate dehydrogenase; PD, progressive disease; SD, stable disease.

### Safety

A total of 4 (40%) of ten patients had injection site reactions deemed related to the ARG1 vaccine. All vaccine-related reactions were < grade 2 according to CTCAE 4.0, and there were no registered vaccine-related Serious Adverse Events or Reactions. All vaccine-related reactions were reversible except for the granuloma formation at injection sites. No patients discontinued the treatment due to injection site reactions. Two (20%) of ten patients had shoulder arthralgia for a few days following vaccination. Six (60%) of ten patients had an increase in transaminases at baseline, and seven (70%) of ten patients had elevated transaminases on treatment. Six (86%) of these seven patients experienced an increase in transaminases during treatment; however, the increase was evaluated as not being vaccine related as they all had a corresponding progression of metastatic lesions in the liver. None of the evaluable patients experienced increased bilirubin levels during treatment. The registered adverse events are listed in **Table 2**.

**Table 2:** A list of registered adverse events.

Adverse Event	*N*	Worst grade according to CTCAE 4.0			
		1	2	3	4
Fatigue	8	5	2	1	0
Granuloma at injection site	4**	1	3	0	0
Rash	2	2	0	0	0
Dry skin	1	1	0	0	0
Nausea	4	2	2	0	0
Constipation	4	4	0	0	0
Vomiting	2	1	1	0	0
Diarrhea	1	1	0	0	0
Ascites	3	1	2	0	0
Dyspnea	2	2	0	0	0
Cough	3	3	0	0	0
Pain	8	3	4	1	0
Arthralgia	2*	2	0	0	0
Infection	1	0	1	0	0
Dizziness	3	3	0	0	0
Xerostomia	1	1	0	0	0
Mucositis	1	0	1	0	0
Alopecia	1	1	0	0	0
Neuropathy	2	2	0	0	0
Hypothyroidism	2	1	1	0	0
Facial paralysis	1	1	0	0	0
Transaminase elevation	7	6	1	0	0
Alkaline phosphate increased	7	5	2	0	0
Anemia	3	0	2	1	0

*possible vaccine-induced adverse events. **vaccine-related adverse events. CTCAE (Common Terminology Criteria for Adverse Events).

### Vaccine responses in blood

PBMCs from patient blood samples were assessed for vaccine specific ARG1 responses using *in vitro* IFNγ ELISPOT assay. Seven (70%) of the ten evaluable patients had evaluable project blood samples at baseline and following a minimum of one evaluation scan. Three (30%) patients only had baseline samples for evaluation (patient number 1, 7, and 12). All three patients refrained from further project blood samples due to progressive disease.

**Figure 2:**
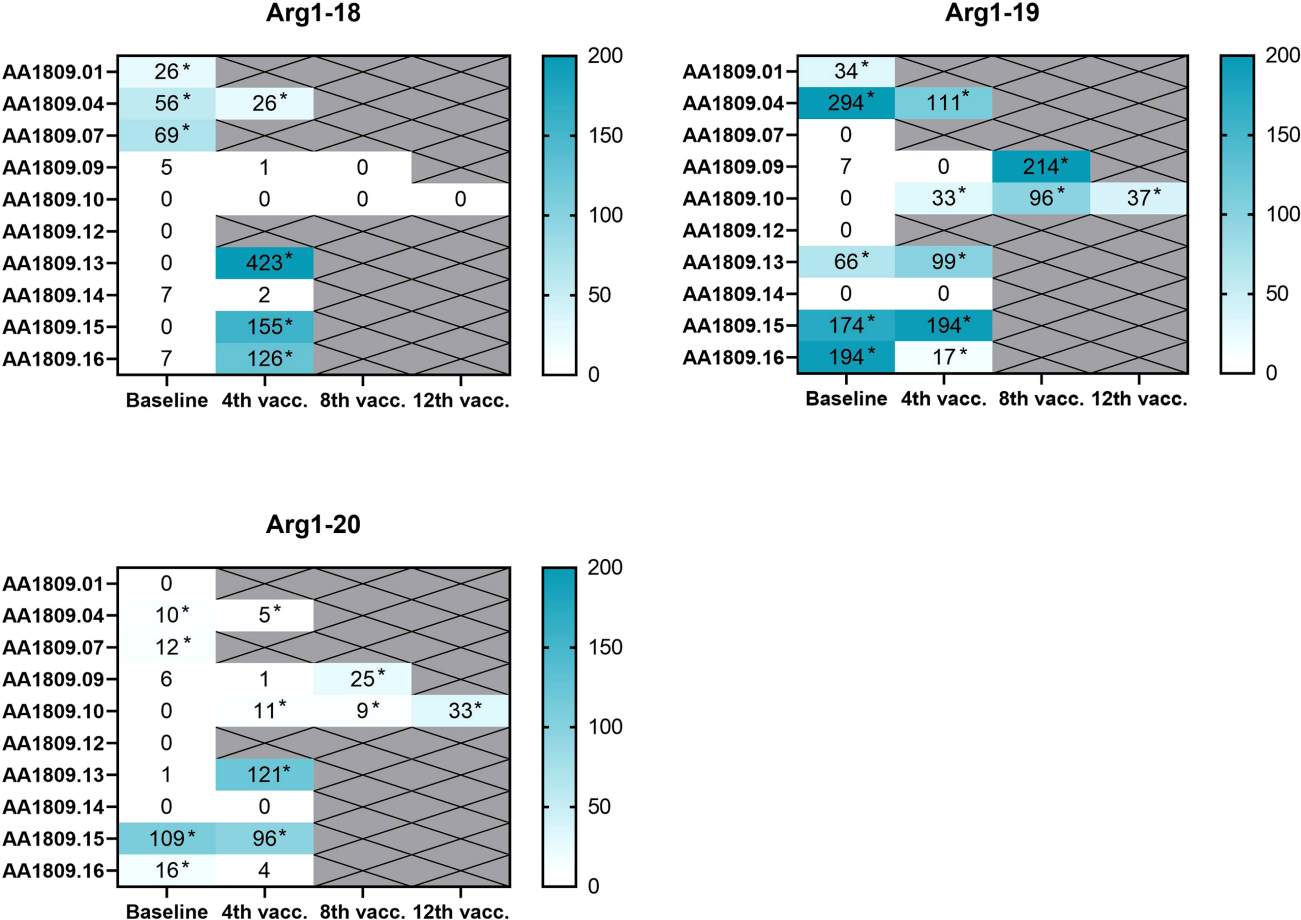
Heatmaps of detected specific arginase-1 (ARG1) responses in peripheral blood mononuclear cells (PBMCs) at baseline and on treatment as measured by interferon (IFN)-γ enzyme-linked immunospot (ELISPOT) assay (n=10). Background has been subtracted. *Indicates positive responses based on Distribution-free Resampling (DFR) method.

Five (50%) of ten patients had pre-existing vaccine-reactive T cells against at least one of the ARG1 peptides at baseline as detected by IFNγ ELISPOT: three (30%) of ten had detectable baseline responses against ARG1-18, five out of ten against ARG1-19, and three (30%) of ten against ARG1-20. Six (86%) of the seven patients evaluable for immune response developed a reactive T cell response against at least one of the ARG1 peptides during treatment. Compared to ARG1 peptide responses detected at baseline, three responses against ARG1-18, three against ARG1-19, and four against ARG1-20 were seen on treatment. Two patients (patient number 9 and patient number 10) with SD on treatment had no T cell response to the ARG1 peptides at baseline; however, both patients developed T cell response to ARG1-19 and ARG1-20 during treatment (**Figure 2**).

**Figure 3:**
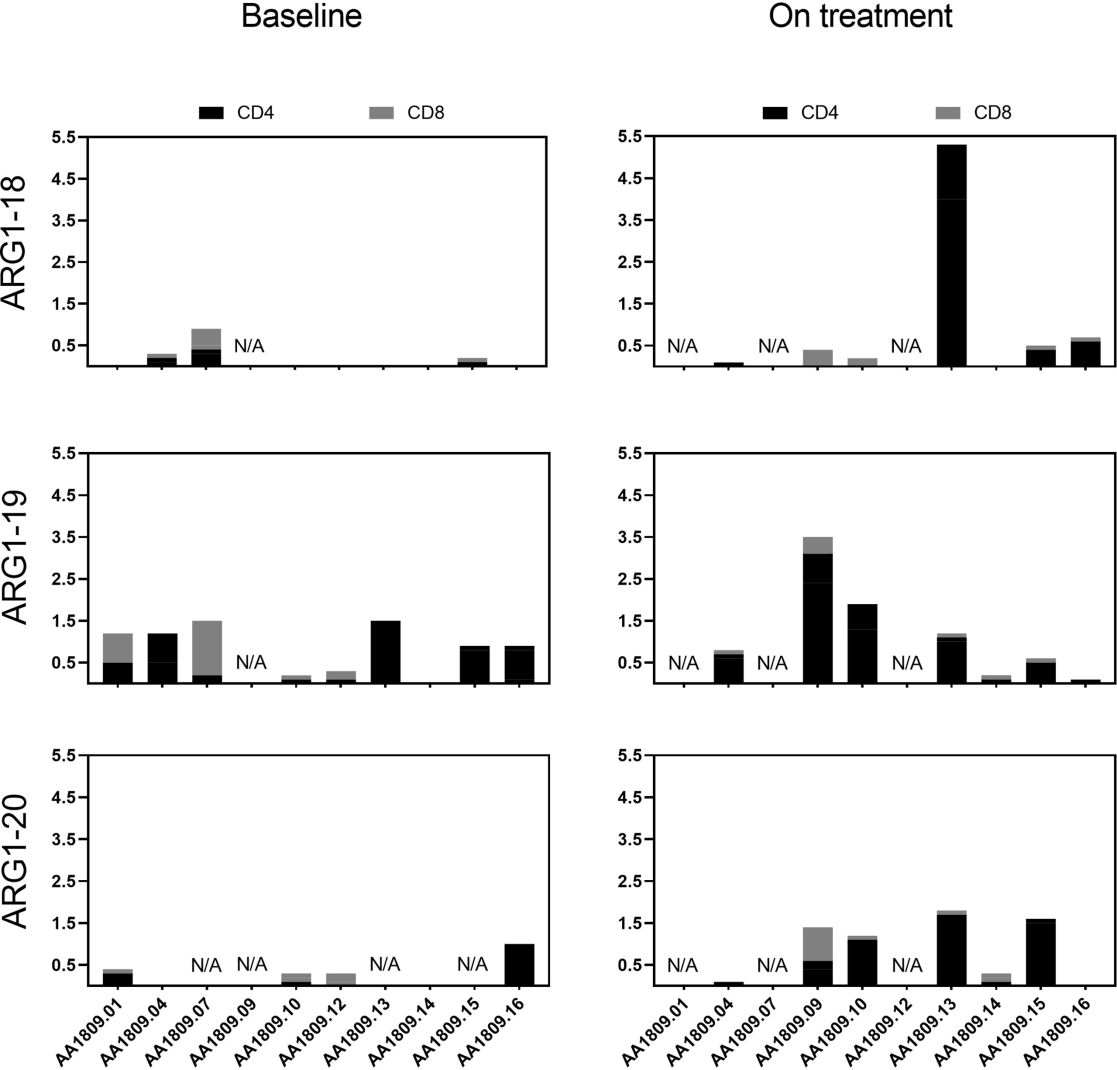
CD4+ and CD8+ arginase-1 (ARG1) vaccine-specific T cell responses in blood. Total ARG1-specific CD4+ (black) and CD8+ (grey) T cell responses in peripheral blood mononuclear cells (PBMCs) at baseline and on treatment. The data were quantified by flow cytometry by an increased expression of interferon (IFN)γ, IFNγ + TNFα, and TNFα after five-hour peptide stimulation. A detailed cytokine expression profile is shown in **Supplementary Figure 1**. The values indicate specific responses subsequent to substraction of background values (n=10). N/A, Not available.

ARG1 peptide responses were additionally characterized using intracellular cytokine staining (ICS) on the *in vitro* stimulated PBMCs. A phenotypic classification revealed that ARG1 vaccine-specific T cells expanded *in vitro* from patients’ PBMCs were both CD8+ T cells and CD4+ T cells (**Figure 3**). Vaccine-specific T cells were shown to produce pro-inflammatory cytokines IFNγ and TNFα in response to ARG1 peptides (**Supplementary Figure 1**)

### Clinical efficacy

**Figure 4:**
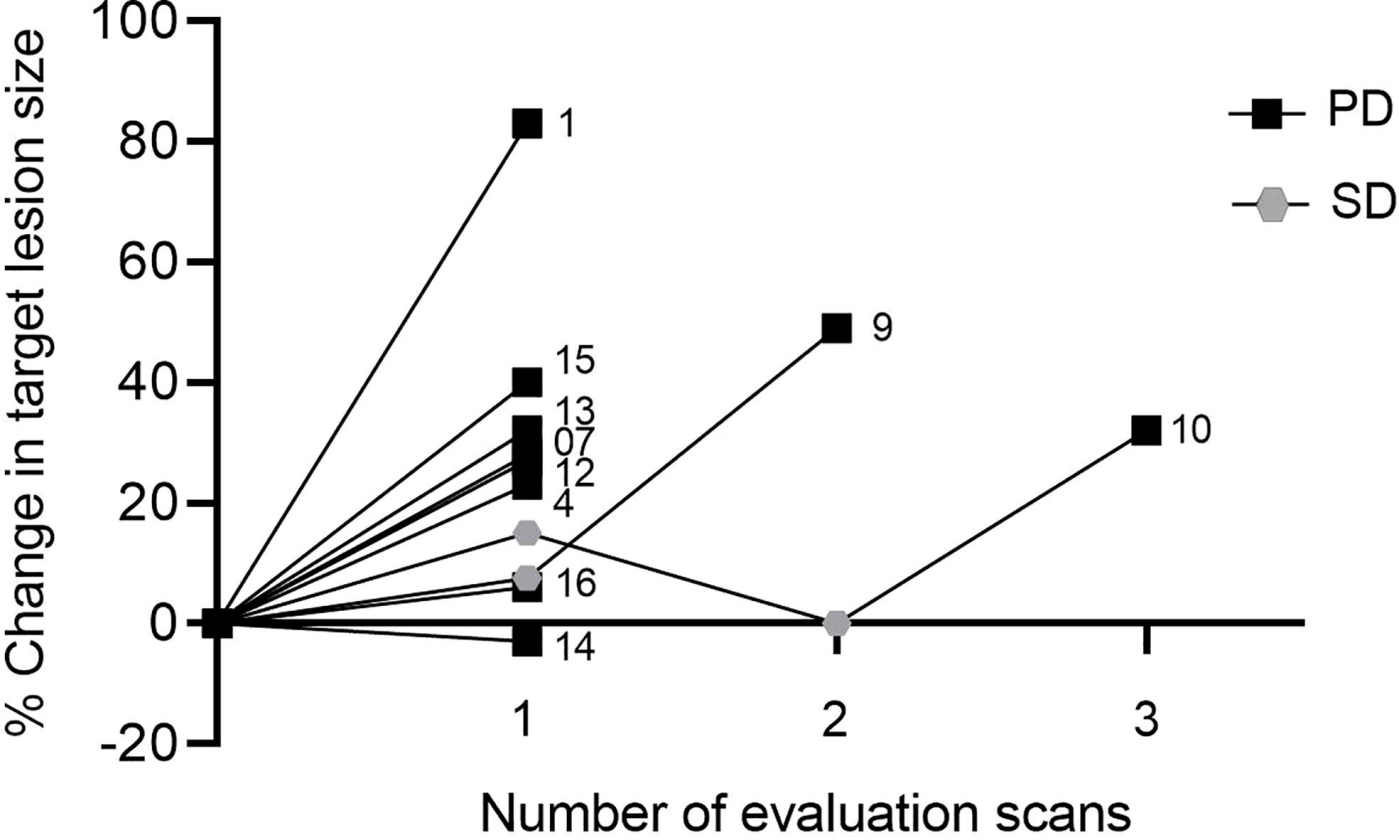
Spider plot showing changes in tumor size on evaluation scans for each patient. Patients were evaluated every three months. Numbers indicate patient ID. SD, stable disease; PD, progressive disease.

At data cut-off, two of ten evaluable patients obtained SD as the best overall response (BOR), and eight patients had PD (**Figure 4**). Patient number 9 was diagnosed with metastatic ocular melanoma in 2018. She had been treated with the checkpoint inhibitor pembrolizumab, followed by chemotherapy with temozolomide before inclusion. BOR to prior treatment was SD on temozolomide. At the first evaluation scan after four ARG1 vaccines, she had SD with an 8% target lesions growth. After the sixth ARG1 vaccine, she experienced clinical progression, and progression was confirmed on a CT scan. Patient number 10 was diagnosed with metastatic melanoma in 2014. Prior to inclusion, he received four treatment lines, including pembrolizumab, temozolomide, re-introduction of pembrolizumab, and the checkpoint inhibitor ipilimumab. BOR before treatment was CR following pembrolizumab. On ARG1 vaccine treatment, the patient had SD at the first and second evaluations scan with a tumor growth of 0%-15%. The patient progressed at the third evaluation scan. Two lesions were irradiated and therefore excluded as target lesions. Patient number 14 had minor target lesion regression with a 3% reduction at the first evaluation scan but developed ascites and was clinically progressing with PS > 2 (**Figure 4**).

**Figure 5:**
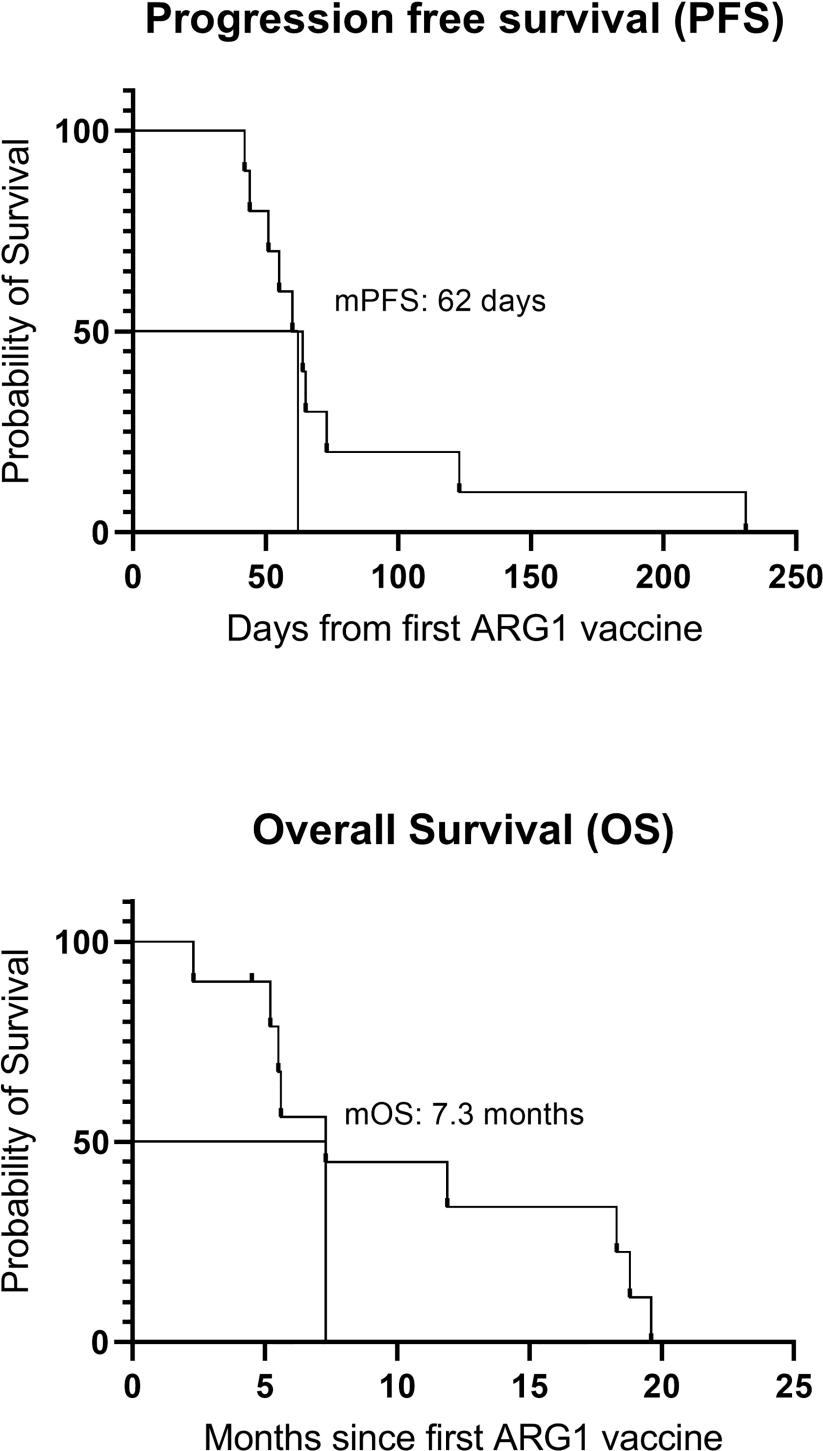
Overall survival and progression-free survival. mPFS, median progression-free survival; mOS, median overall survival.

Median progression-free survival (mPFS) was 62 days, and median overall survival (mOS) from the time of the first vaccine was 7.3 months (**Figure 5**).

## Discussion

In this clinical phase I study, ten patients with metastatic solid tumors were treated with a peptide vaccine targeting ARG1. The primary objective was to assess the vaccine safety, and we found that the ARG1 vaccine was well tolerated. We did not observe any grade 3–4 vaccine-related adverse events according to CTCAE 4.0 (**Table 2**). In line with the previous similar vaccination trials targeting self-proteins expressed by regulatory immune cells conducted at CCIT-DK, the most frequent vaccine-induced adverse event was injection site reactions (17–19). Because ARG1 is expressed in the liver and other non-cancerous tissues, there was a risk of inducing autoimmune reactions. We found that only patients with progressing metastatic lesions in the liver experienced increased transaminases during treatment. Another clinical trial additionally examined an oral ARG1 inhibitor (CB-1158), and the treatment did not induce significant on-target toxicity (24).

Notably, an ELISPOT-based evaluation of the immunogenicity of the ARG1 vaccine revealed that nine (90%) of ten evaluable patients had peptide-specific T cell responses against minimum one of the ARG1 peptide sequences in the blood and six (86%) of the seven evaluable patients developed a reactive T cell response against at least one of the ARG1 peptides during treatment. The ARG1-specific T cells comprised both CD4+ and CD8+ cells, although CD4+ reactivity was most frequent and of higher magnitude. Evaluation scans revealed two patients obtained SD on vaccination treatment, and eight had PD. The trial was, however, not designed to evaluate clinical efficacy. At inclusion, all patients had progressive advanced cancer disease and had received multiple lines of therapy (**Table 1**). Consequently, we did not expect a monotherapeutic ARG1 peptide vaccine to induce objective responses.

The trial was limited by the small sample size and the low number of vaccine administrations. Including patients across diagnoses made the results difficult to compare as well. Despite excluding patients with performance status >1 and short life expectancy, three patients received less than two ARG1 vaccines and were replaced with new participants due to fast disease progression. Including patients with SD off standard treatment could potentially have increased the number of vaccine administrations. However, this patient group is difficult to recruit because most patients with SD still receive maintenance therapy or have paused treatment due to adverse reactions. Despite the limitations, this clinical trial was valuable for assessing the safety and immunogenicity of a peptide vaccine targeting ARG1. A clinical trial has already been initiated based on early ARG1 safety data (NCT04051307).

Peptide-based cancer vaccines are well tolerated in general, but due to their limited potency, they are unlikely to succeed as a monotherapy for patients with metastatic tumors (25). The future role of an ARG1-based cancer vaccine is rather in an adjuvant setting or in combination with other immunotherapeutic treatment modalities. The infiltration of TAMs in tumors correlates with a poor prognosis and a poor response to therapies, including CPI therapies (26). The combination of ARG1-based therapeutic vaccines and CPIs may therefore be especially attractive since ARG1-specific T cells can directly target immunosuppressive TAMs and thereby induce Th1inflammation in the TME. This can further induce the expression of proteins like PD-L1 in different cell types in the TME. ARG1-based immunomodulatory vaccination aims to convert the immune hostile TME and generate targets more disposed to anti-PD-1/PD-L1 immunotherapy. Hence, ARG1-based immune vaccines that modulate the tumor microenvironment should increase the effect of CPIs (13). To support this, we recently showed the anti-tumor effects of ARG1-based vaccination in several different murine cancer models. ARG1-vaccination activated peptide-specific T cells and induced tumor growth control upon vaccination (27). Importantly, ARG1-based vaccination indeed functions in synergy with anti-PD-1 therapy in these models. ARG1-targeting therapeutic vaccines changed the cell composition of the TME, resulting in increased T cell infiltration and a change in the M1/M2 ratio of tumor-infiltrating macrophages. In addition, we observed decreased ARG1 expression and a reduced suppressive function of tumor-educated myeloid cells following ARG1 vaccination (27). Hence, the combination therapy of ARG1-based vaccines and CPIs could increase the number of patients responding to therapy. In line with the pre-clinical data at CCIT-DK, we recently obtained impressive response and survival rates in metastatic melanoma by combining an immune modulatory peptide vaccine targeting IDO and PD-L1 with the CPI nivolumab in a phase I–II clinical trial (NCT03047928) (17).

## Conclusion

This trial was a small pilot study conducted to establish the initial proof of safety and immunogenicity of an ARG1 peptide vaccine. This study showed that the ARG1 peptide vaccine was safe, and the vaccine administration was feasible. Clinical responses were limited, but the vaccine induced an immune response in the majority of patients. In combination with other immunotherapies, the ARG1 cancer vaccine could likely play a role in future cancer treatment strategies for patients with high levels of ARG1-expressing cells in the TME.

## Data availability statement

The original contributions presented in the study are included in the article/**Supplementary Material**. Further inquiries can be directed to the corresponding author.

## Ethics statement

The studies involving human participants were reviewed and approved by the committees on health research ethics in the Capital Region of Denmark, Regionsgården. The patients/participants provided their written informed consent to participate in this study. Written informed consent was obtained from the individual(s) for the publication of any potentially identifiable images or data included in this article.

## Author contributions

CL gained institutional approval, performed the research, and wrote the manuscript. CL and JK recruited, evaluated, and treated the patients. SM and RH evaluated and treated the patients. IS, MA, EE, AP, EM, and RH critically revised the manuscript. EM performed laboratory research and data analysis and critically revised the manuscript. MA, IS, and EE conceptualized and designed the trial and supervised the project. All authors contributed to the article, and all authors approved the versions for submission.

## Funding

The trial was funded by Herlev Hospital and through a research funding agreement between the Oncology Department at Herlev Hospital, National Center for Cancer Immune Therapy (CCIT-DK), and IO Biotech ApS.

## Acknowledgments

We express our gratitude to the trial patients for the participation. We thank the laboratory technicians from CCIT-DK for technical support. We thank L. Sengeløv, the head of the Oncology Department at Herlev Hospital, and the nurses at Clinic 5.

## Conflict of interest

MA has various patent applications in relation to the therapeutic uses of ARG1 peptides. The patents are allocated to the company IO Biotech. MA is a founder, advisor, and shareholder for IO Biotech. EM, AP, and EE are employees at IO Biotech. IS has lectured for or had advisory board relationships with MSD, Sanofi Aventis, BMS, Pierre Fabre, Novartis, TILT Biotherapeutics, IO Biotech, and Novo Nordisk. IS has received research grants from Lytix biopharma, IO Biotech, BMS, Adaptimmune, and TILT Biotherapeutics. IS is a co-founder and shareholder for the company IO Biotech.

The remaining authors declare that the research was conducted in the absence of any commercial or financial relationships that could be construed as a potential conflict of interest.

## Publisher’s note

All claims expressed in this article are solely those of the authors and do not necessarily represent those of their affiliated organizations, or those of the publisher, the editors and the reviewers. Any product that may be evaluated in this article, or claim that may be made by its manufacturer, is not guaranteed or endorsed by the publisher.
