# Supplementary material for: Arginase-1 targeting peptide vaccine in patients with metastatic solid tumors – A phase I trial
Source: Front Immunol. 2022 Oct 17;13:1023023. doi: 10.3389/fimmu.2022.1023023 (PMC9622376; doi:10.3389/fimmu.2022.1023023)
Supplement: Supplementary file 1 [file DataSheet_1.pdf]

Supplementary figure 1

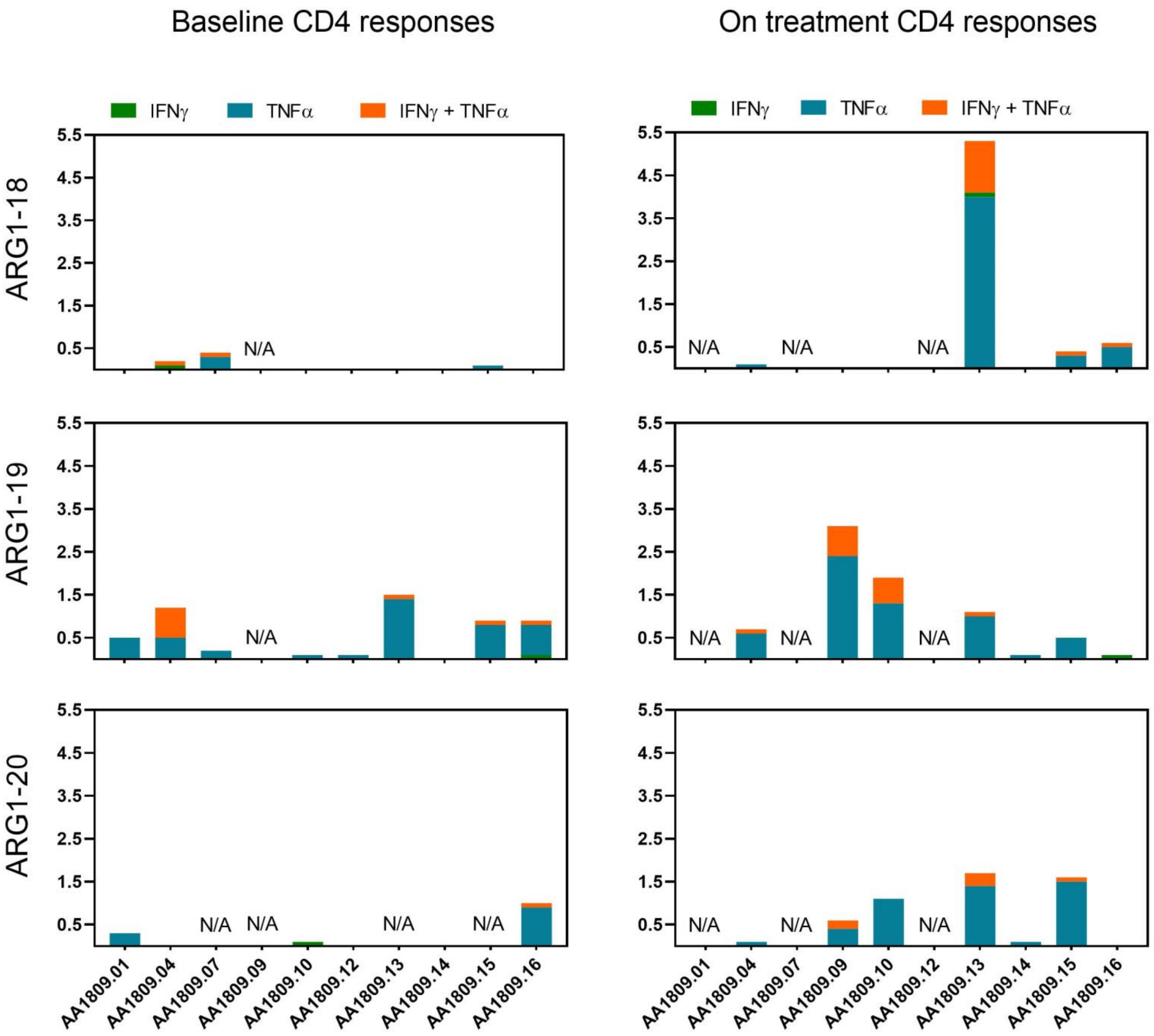

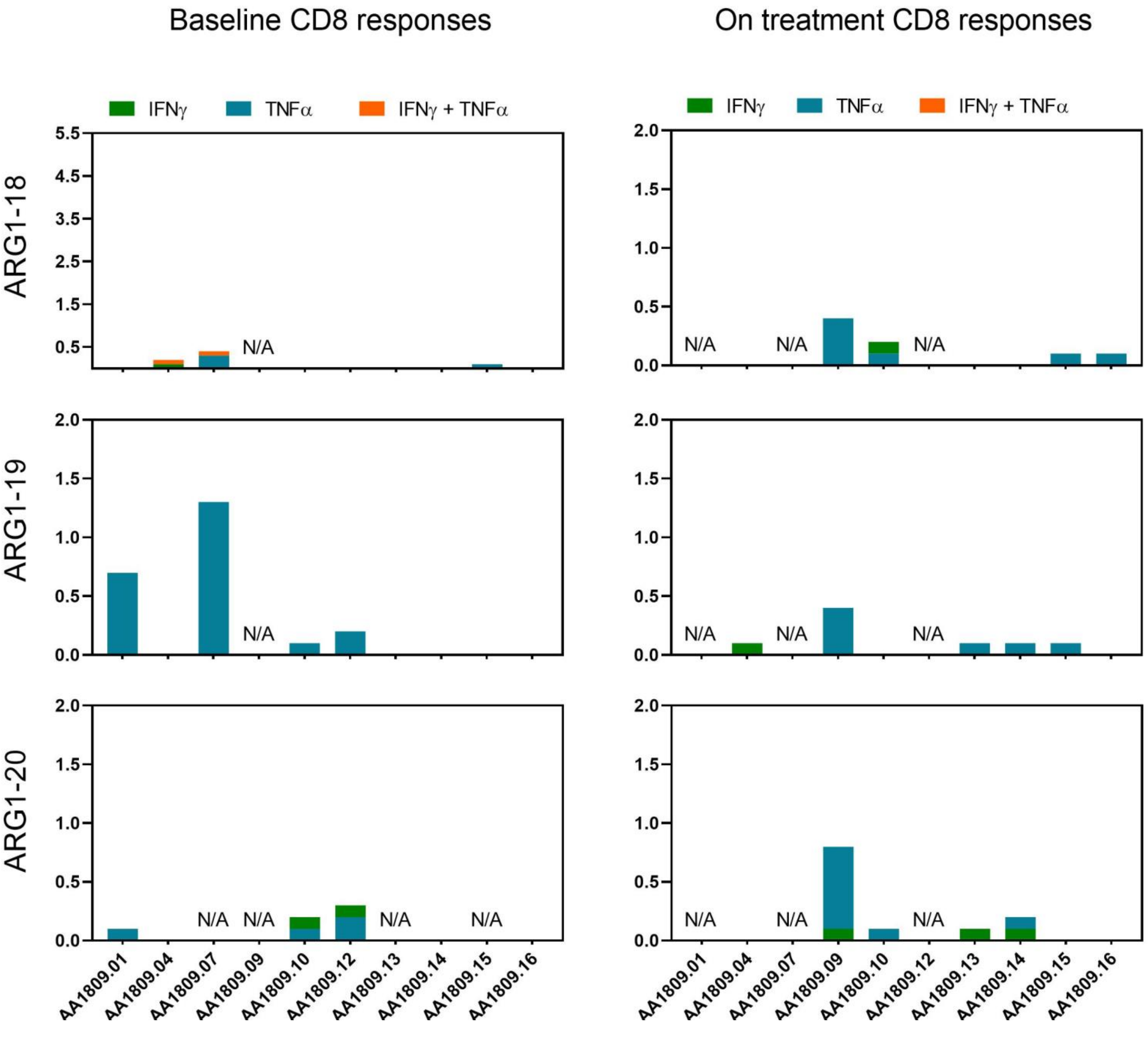

Supplementary figure 1: CD4+ and CD8+ arginase-1 (ARG1) vaccine-specific T cell responses in blood. Top: ARG1-specific CD4+ T cell responses in peripheral blood mononuclear cells (PBMCs) at baseline and on treatment. Bottom: ARG1-specific CD8+ T cell responses in PBMCs at baseline and on treatment. The data were quantified by flow cytometry by an increased expression of interferon (IFN)γ, IFNγ + TNFα, and TNFα after five-hour peptide stimulation. The values represent specific responses subsequent to the substraction of the background values (n=10).
